# Effects of Different Communication Tools on the Efficiency of Anesthesiologists in the Perioperative Setting

**DOI:** 10.4172/2155-6148.1000794

**Published:** 2017-12-21

**Authors:** Cindy Yeoh, Kay See Tan, Jennifer Mascarenhas, Luis Tollinche

**Affiliations:** Department of Anesthesiology and Critical Care Medicine, Memorial Sloan Kettering Cancer Center, New York, USA

**Keywords:** Real time locating systems (RTLS), Wireless LAN-based interactive voice response systems (IVR), Communication tools, Healthcare providers, Anesthesiologists, Perioperative efficiency, Workflow

## Abstract

**Objective:**

To assess if Real Time Locating Systems (RTLS) technology has an effect on the perioperative efficiency of anesthesiologists at our institution.

**Methods:**

A retrospective chart review was performed for all outpatient and short-stay patients who received general anesthesia and monitored anesthesia care between January and June of 2016. Patients over 18 years with an ASA classification of 1, 2, and 3 were included. Time was used as a measure of efficiency between two groups of anesthesiologists.

These two comparison groups were as follows:

**Group 1:** Anesthesiologists at the academic center’s main campus who do not have access to RTLS
**Group 2:** Anesthesiologists at Josie Robertson Ambulatory Surgical Center where RTLS is available and use of RTLS is compulsory

Two outcome measures were collected from patient electronic records:

1. **DUR1:** Duration between when patient is admitted to a presurgical bed and preoperative evaluation by the attending anesthesiologist
2. **DUR2:** Duration between when patient is admitted to the operating room and initiation of induction by the attending anesthesiologist.

**Results:**

Anesthesiologists who had access to RTLS technology were found to be more efficient in completing their preoperative anesthesia evaluation and initiating intraoperative induction. They took less time to complete these tasks and the difference was statistically significant to p<0.0001

**Conclusion:**

Anesthesiologists at our institution, who have access to RTLS as an additional communication tool, were found to be consistently more efficient in their perioperative workflow. There are confounding factors that can account for the shorter times and more efficient perioperative workflow of anesthesiologists. With continued application and investigation over time, the utility of RTLS on workflow efficiency of healthcare providers will become more apparent.

## Background

Historically, common modes of communication within healthcare systems have included the telephone and pager. More recently, in addition to an increased use of personal cell phones in the healthcare setting, innovation in telecommunications has led to the introduction of more advanced systems such as wireless LAN-based interactive voice response systems (IVR) and real-time locating systems (RTLS).

IVR allows employees to interact with the hospital’s host system via a telephone keypad or by speech recognition to service their own inquiries through IVR dialogue. RTLS is a technology that allows tracking of assets and individuals within a hospital and has the potential for many applications in healthcare [1].

While both systems are gaining traction in healthcare settings, neither system has been widely implemented to date [1]. However, at our institution, wireless LAN-based IVR system has long been in use, and RTLS was recently implemented in the new ambulatory surgical center when the center became operational in January 2016.

Due to the existence of such varied options in healthcare communication tools, we investigated the effectiveness of our communication tools. The purported benefit of RTLS includes higher efficiency work flow (1). In the healthcare setting, higher efficiency includes quicker turnover time between surgical cases in the operating room. The anesthesia team is a critical component of ensuring efficient turnover [2,3].

In addition to delivering safe care to their patient, the anesthesiologist is charged with securing operating room efficiency. In fact, at most hospitals in the US, operating rooms staffing and turnover decisions are led by a member of the anesthesia team [4]. It has been reported that every min of operating room time runs between $30 and $80 per min [5,6].

The operating room requirement of highly specialized teams and intensive resources renders it the single most expensive unit in hospitals [7]. This cost disparity has driven the abundance of literature dedicated to improving operating room efficiency [7].

Several time points have been identified where potential savings may occur. Among these is the time squandered when the patient is being prepared for induction of anesthesia. In most hospitals, the patient is escorted to the operating room by a member of the perioperative team who is not the anesthesiologist that is required to be in the room for induction.

This results in unnecessary delays as the operating room is ‘on hold’ pending the arrival of the anesthesiologist. It follows, then, that efficiency would improve if we could reduce this waiting time [2,3].

Another area where reduced waiting time may be easily decreased is the preoperative visit. Anesthesiologists are required to conduct a preoperative visit in advance of taking patients to the operating room. This visit is typically conducted in a dedicated presurgical area. Oftentimes, the operating room is prepared and waiting for a patient whose anesthesiologist has not completed the preoperative visit [2,3].

RTLS may decrease these waiting times in one of two ways:

1. Operating room staff is able to track the whereabouts of the entire OR team (including the patient) allowing for more efficient turnover process when the OR is deemed ready.
2. Providers may display behavioral modifications that improve efficiency. Knowing that their whereabouts are being tracked creates motivation for anesthesiologists to be readily available when their presence is requested [1].

In our study we investigated two time points during the anesthesiologist workflow to determine if any difference exists in efficiency among anesthesiologists who are using RTLS.

## Methods

A retrospective chart review was performed for all outpatient and short-stay patients who received general anesthesia (GA) and monitored anesthesia care (MAC) between January 2016 and June 2016. Patients over 18 years with an ASA physical status classification of 1, 2 and 3 were included. Time was used as a measure of efficiency between two groups of anesthesiologists.

The two comparison groups were as follows:

**Group 1:** Anesthesiologists at the academic center’s main campus who do not have access to RTLS
**Group 2:** Anesthesiologists at Josie Robertson Ambulatory Surgical Center where RTLS is available and use of RTLS is compulsory

Both sites utilize wireless LAN-based IVR and clinicians frequently depend on other communication tools including mobile phones, text paging and land line.

To ensure comparable patient case-mix between the two groups, the criteria of inclusion were those given GA or MAC, outpatient and short-stay, and ASA 1, 2, or 3.

Two outcome measures were collected from patient electronic records:

1. **DUR1:** Duration (in min) between when patient is admitted to a presurgical bed and preoperative evaluation by the attending anesthesiologist
2. **DUR2:** Duration (in min) between when patient is admitted to the operating room and initiation of induction by the attending anesthesiologist.

The outcomes were summarized as medians (25^th^, 75^th^ percentile) for each group (Main Campus *vs*. Josie Robertson) and compared between the groups using the Wilcoxon Rank Sum test (stratified by the ASA score).

Institutional protocol requires evaluation of first case of the day to be completed by 7:15 am. Hence, as exploratory analyses, we also summarized outcomes by group to detect if the outcomes were sensitive to the time of surgery: (a) first case of the day *vs*. (b) non-first case of the day.

## Results

Data was drawn from retrospective review of 8584 patient electronic records. Records included were patients over 18 years, GA (N=6299) and MAC (N=2285), outpatient (N=5718) and short stay (N=2866), and ASA classification 1, 2 and 3. Of these, 3880 records were GA cases performed at Josie Robertson, while 2419 were cases performed at Main Campus.

77 records were excluded due to data error (e.g. negative time values were recorded because of electronic error involving clearing of patients before their arrival to the presurgical area).

**Figure 1:**
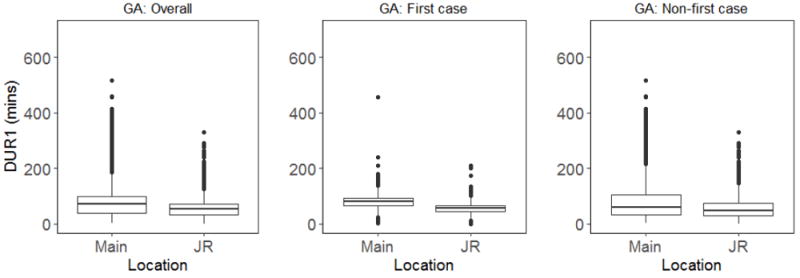
Box plot comparison of DUR1 for all GA cases. GA: General anesthesia; DUR1: Duration (in minutes) between when patient is admitted to a presurgical bed and preoperative evaluation by the attending anesthesiologist; Main: Main Campus; JR: Josie Robertson Ambulatory Surgical Center.

For the first outcome measure, time to anesthesia preoperative clearance, data analysis for all general anesthesia cases (first case of the day+non-first case of the day, N=6248) showed that anesthesiologists in Group 1 took significantly more time to evaluate patients than anesthesiologists in Group 2. The median duration was 72 min in Group 1 versus 52 min in Group 2 (p value of <0.0001) (Table 1 and Figure 1).

Subanalysis of “first case of the day” data also showed a significant difference in time taken to evaluate patients between the two groups of anesthesiologists. Anesthesiologists in Group 1 took a median time of 81 min, while those in Group 2 took 56 min (p value <0.0001) (Table 2 and Figure 1).

Similarly, for all “non-first” cases, median time for Group 1 anesthesiologists to evaluate patients after admission to a presurgical bed was 59 min compared to 48 min for anesthesiologists in Group 2 (p<0.0001) (Table 3 and Figure 1).

**Figure 2:**
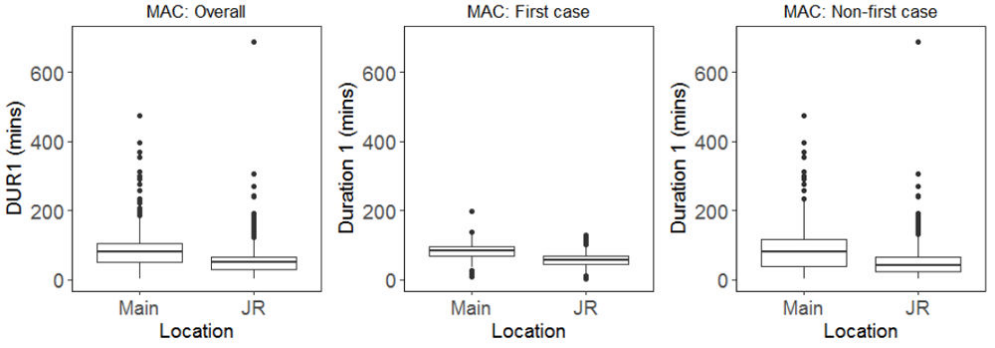
Box plot comparison of DUR1 for all MAC cases. MAC: Monitored anesthesia care; DUR1: Duration (in minutes) between when patient is admitted to a presurgical bed and preoperative evaluation by the attending anesthesiologist; Main: Main Campus; JR: Josie Robertson Ambulatory Surgical Center.

Results were similar when data for all MAC cases (first case and non-first case, N=2259) was analyzed. Median time for anesthesiologists in Group 1 was 81 min versus 50 min for Group 2 (p<0.0001) (Table 4 and Figure 2).

When data was subdivided and analyzed by first case versus nonfirst case MAC procedures, a similar trend was observed with Group 1 anesthesiologists taking 82 min *vs*. 57 min for Group 2 (first case only, p<0.0001) and Group 1 anesthesiologists taking 79 min *vs*. 42 min for Group 2 (non-first case, p<0.0001) (Tables 5 and 6 and Figure 2).

**Figure 3:**
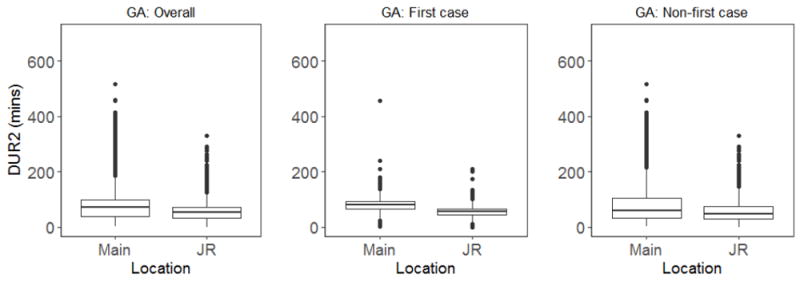
Box plot comparison of DUR2 for all GA cases. GA: General anesthesia; DUR2: Duration (in minutes) between when patient is admitted to the operating room and initiation of induction by the attending anesthesiologist; Main: Main Campus; JR: Josie Robertson Ambulatory Surgical Center.

For the second outcome measure, time to anesthesia induction, data analysis once again showed a statistically significant difference in time to anesthesia induction between the two groups. For all general anesthesia cases (N=6265), Group 1 had a median time of 9 min versus 8 min for Group 2 (p<0.0001) (Table 7 and Figure 3).

All general anesthesia cases were further analyzed according to first case versus non-first case, and the same trend was observed for both groups. For first case of the day, Group 1 anesthesiologists took a median of 10 min for induction after patient arrival to the OR versus 8 min for Group 2 (p<0.0001) (Table 8 and Figure 3). For non-first case of the day, Group 1 anesthesiologists took a median of 9 min versus 8 min for Group 2 (p<0.0001) (Table 9 and Figure 3).

## Discussion

RTLS is a technology that allows tracking of assets and/or persons in real time. While still considered early in its development, application, and implementation, it has established utility in various industries including healthcare [1,8,9]. Monitoring patient flow and tracking of hospital assets/equipment allows for diagnosis of existing problems that can significantly improve work flow and patient care when corrected [9]. There are many reports of RTLS-enabled decrease in hospital wait times and length of stay for patients, which translate to increased patient satisfaction [10,11]. These are proven benefits of RTLS.

The same concept of RTLS-enabled enhanced work flow and increased productivity is also the basis for expanding use of the technology to include healthcare providers such as physicians and nurses.

This technology is still relatively new at our institution and is only available at one of our facilities- the Josie Robertson Ambulatory Surgical Center (JRSC). This center became operational in January of 2016 and has utilized RTLS since its opening. All individuals, patients and healthcare providers, are required to wear RTLS tracking devices while at the center. The purpose of our study was to determine if any difference exists in the perioperative efficiency of anesthesiologists at JRSC compared to Main Campus. We were interested in two outcome measures, time to anesthesia clearance (after patient is admitted to a presurgical bed) and time to anesthesia induction (after patient arrives to the operating room).

Data analysis showed consistently shorter times for both outcome measures for anesthesiologists in the JRSC group (Group 2). While this can be attributed to the presence of RTLS at Josie Robertson Ambulatory Surgical Center, there are confounding factors that could account for the difference in perioperative efficiency of the anesthesiologists.

Patients across groups were matched by ASA classification and complexity of case as determined by same day/short stay cases. However, only ambulatory MAC and GA cases of low complexity are done at JRSC. As a result, operating room turnover time tends to be shorter and more predictable at JRSC compared to Main Campus, where complicated cases with sicker patients often translates into unpredictably longer operating times. Because of this, anesthesiologists at Main Campus might not see the need to perform their preoperative evaluation as soon as a patient arrives at a presurgical bed. Similarly, the complexity of cases performed at Main Campus could also explain why the duration to anesthesia induction is longer than that at JRSC. If an anesthesiologist is involved in starting a complicated case, his or her response to a call for induction in a second concurrent room will be delayed. Faster response times of anesthesiologists at JRSC could also be attributed to positive behavior modification as a consequence of personnel tracking.

We analyzed first cases of the day versus non-first cases separately, expecting data to show that first cases would have shorter times to anesthesia clearance because of an institutional policy requiring all first cases to be evaluated by anesthesiologists before 7:15 am. However, our data showed that duration to anesthesia clearance was in fact longer for first cases of the day for all case types, MAC and general anesthesia. This could be attributed to varying arrival times of anesthesiologists to the workplace, combined with extremely early arrival times of patients who are first cases of the day to the presurgical area.

Future studies should be conducted to investigate RTLS efficiencies while excluding these possible confounders. The ideal study will analyze an institution’s efficiency pre and post RTLS implementation to control for these variables. Additional work in this area will also include expansion of time metrics to focus on other perioperative clinician’s workflow (nursing and surgery).

## Conclusion

Anesthesiologists at our institution, who utilize RTLS as an additional communication tool, were found to be consistently more efficient in their perioperative workflow. They had faster time to preoperative anesthesia clearance and shorter duration to anesthesia induction upon patient arrival to the operating room. There are confounding factors that can account for the shorter times and more efficient perioperative workflow of anesthesiologists. With continued application and investigation over time, the utility of RTLS on workflow efficiency of healthcare providers will become more apparent.

## Figures and Tables

**Table 1:** GA: Overall (First case+Non-first case), median (25th, 75th percentile).

	MAIN CAMPUS (N=2419; 38%)	JOSIE ROBERTSON (N=3880; 62%)	p
DUR1 (N=6248)	72.0 (39.0, 97.0)	52.0 (33.0, 70.0)	<0.0001

GA: General Anesthesia; DUR1: Duration (in min) between when patient is admitted to a presurgical bed and preoperative evaluation by the attending anesthesiologist.

**Table 2:** GA: First case.

	MAIN CAMPUS (N=892; 38%)	JOSIE ROBERTSON (N=1432; 62%)	p
DUR1 (N=2311)	81.0 (65.0, 93.0)	56.0 (44.0, 66.0)	<0.0001

GA: General Anesthesia; DUR1: Duration (in min) between when patient is admitted to a presurgical bed and preoperative evaluation by the attending anesthesiologist.

**Table 3:** GA: Non-first case.

	MAIN CAMPUS (N=1527; 38%)	JOSIE ROBERTSON (N=2448; 62%)	p
DUR1 (N=3937)	59.0 (32.0, 105.0)	48.0 (28.0, 75.0)	<0.0001

GA: General Anesthesia; DUR1: Duration (in min) between when patient is admitted to a presurgical bed and preoperative evaluation by the attending anesthesiologist.

**Table 4:** MAC: Overall (First case+Non-first case).

	MAIN CAMPUS (N=374; 16%)	JOSIE ROBERTSON (N=1911; 84%)	p
DUR1 (N=2259)	81.0 (49.0, 104.0)	50.0 (29.0, 66.0)	<0.0001

MAC: Monitored anesthesia care; DUR1: Duration (in min) between when patient is admitted to a presurgical bed and preoperative evaluation by the attending anesthesiologist.

**Table 5:** MAC: First case.

	MAIN CAMPUS (N=115; 15%)	JOSIE ROBERTSON (N=666; 85%)	p
DUR1 (N=776)	82.0 (68.0, 95.0)	57.0 (45.0, 67.0)	<0.0001

MAC: Monitored anesthesia care; DUR1: Duration (in min) between when patient is admitted to a presurgical bed and preoperative evaluation by the attending anesthesiologist.

**Table 6:** MAC: Non-first case.

	MAIN CAMPUS (N=259; 17%)	JOSIE ROBERTSON (N=1245; 83%)	p
DUR1 (N=1483)	79.0 (37.0, 115.0)	42.0 (23.0, 66.0)	<0.0001

MAC: Monitored anesthesia care; DUR1: Duration (in min) between when patient is admitted to a presurgical bed and preoperative evaluation by the attending anesthesiologist.

**Table 7:** GA: Overall (First case+Non-first case).

	MAIN OR (N=2419; 38%)	JOSIE ROBERTSON (N=3880; 62%)	p
DUR2 (N=6265)	9.0 (7.0, 12.0)	8.0 (6.0, 10.0)	<0.0001

GA: General Anesthesia; DUR2: Duration (in min) between when patient is admitted to the operating room and initiation of induction by the attending anesthesiologist.

**Table 8:** GA: First case.

	MAIN OR (N=892; 38%)	JOSIE ROBERTSON (N=1432; 62%)	p
DUR2 (N=2314)	10.0 (7.0, 14.0)	8.0 (6.0, 10.0)	<0.0001

GA: General Anesthesia; DUR2: Duration (in min) between when patient is admitted to the operating room and initiation of induction by the attending anesthesiologist.

**Table 9:** GA: Non-first case.

	MAIN OR (N=1527; 38%)	JOSIE ROBERTSON (N=2448; 62%)	p
DUR2 (N=3951)	9.0 (6.0, 12.0)	8.0 (6.0, 10.0)	<0.0001

GA: General Anesthesia; DUR2: Duration (in min) between when patient is admitted to the operating room and initiation of induction by the attending anesthesiologist.
